# Psychiatry: Past, Present and Prospect

**DOI:** 10.1192/pb.bp.114.048413

**Published:** 2015-04

**Authors:** Elaine Murphy

**Figure:**
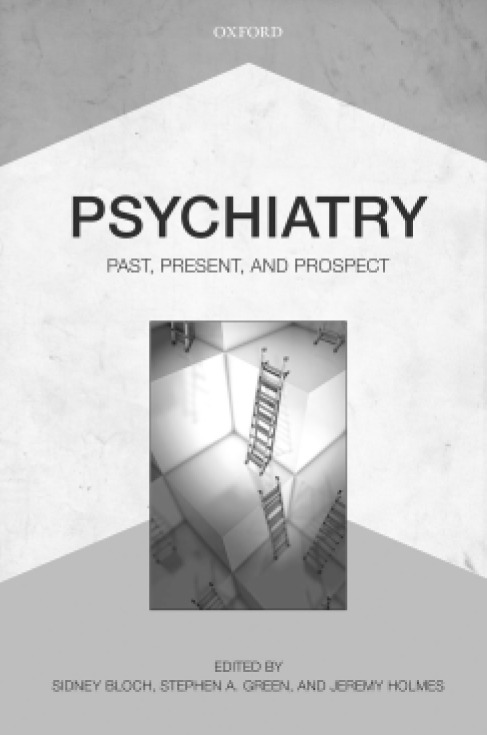


The editors of this prodigiously ambitious collection of personal essays hint that they strived with some difficulty to extract from their distinguished authors personal views of how psychiatry has developed during the course of the past 50 years. The editors did not want a series of academic reviews but more the experience and wisdom gleaned during successful careers. This is history but as reminiscence, memoir as well as critical scientific review. The editors have achieved magnificently what they set out to do and nearly all the individual essays are worth spending time absorbing. The book is quite simply the best account, a searingly honest one, of the progress we have made over the working lifetime of the internationally renowned authors of the essays, nearly all like your reviewer at the tail end of their careers.

Every practising psychiatrist and trainee should read this book. I did not just read it, I devoured it, but readers beware, I put down the finished book with a disturbing sense of disappointment. Have we really changed the prospects for our patients’ lives so little in 50 years? Has so much neuroscientific research, psychopharmacology, sociology and changing political environments produced so little? I fear so. ‘What’s past is prologue’, as pronounced by Shakespeare in *The Tempest*. The real story is yet to be revealed.

The perennial indecisiveness about the boundaries of psychiatry’s responsibilities, the repeated creation of social movements that ultimately fail to shift patients’ life chances, the deficiency in translating what we know from social psychiatric studies into practical treatment modalities, the ever-shifting ethical sands of risk and restraint and the almost total lack of significant improvement in medications after imipramine in 1940 and clozapine in the 1960s, all this makes disturbing reading. Yet many essays contain scholarly reviews of fruitful paths of research that have not quite yielded success yet, such as Peter McGuffin on genetics, Steven Hyman on neuroscience, Edwin Harari on personality disorders. We are always on the brink, looking upwards but not quite over the brow of the hill just yet. Inevitably, the reader is drawn to areas of one’s own personal interest. This reviewer turned straight away to George Szmukler’s entertaining review of the vicissitudes of legal controls versus professional judgement, Paul Mullen and Danny Sullivan’s pithy and sceptical account of the development of forensic psychiatry and Julian Leff’s *cri de coeur* bemoaning the loss of social psychiatry developments from its exciting origins. Why have we not implemented what we know from social skills training and education for patients with schizophrenia, for example? I fear the answer is that mental health services have plenty of doctors and nurses but insufficient numbers of educationalists, social work interventionists and behavioural trainers. The administrative context in which psychiatry is practised has remained almost unchanged in the past 50 years, asylums are gone but the care and treatment has barely changed. Scientific endeavour plods on, our understanding of aetiology makes modest progress, but clinical practitioners must do the best they can with inadequate tools, today just as our colleagues did 50 years ago.

I have one criticism and this is not of the authors or editors. Oxford University Press should surely have produced this book in a better-quality format. It is printed in a small font (although not as small as this journal!) and the cover is somewhere between dull and unfathomable; it looks cheap. A tome so rich in content deserves a more sumptuous coat.

